# Gender difference and effect of pharmacotherapy: findings from a smoking cessation service

**DOI:** 10.1186/s12889-016-3672-y

**Published:** 2016-10-03

**Authors:** N. J. Walker, H. C. van Woerden, V. Kiparoglou, Y. Yang, H. Robinson, E. Croghan

**Affiliations:** 1 Oxford Biomedical Research Centre, Churchill Hospital, Oxford, England; 2 Institute of Primary Care & Public Health, Cardiff University, Cardiff, Wales; 3 Centre for Health Sciences, University of the Highlands and Islands, Inverness, Scotland; 4 Nuffield Department of Primary Care Health Science, University of Oxford, Oxford, England; 5 Quit 51 Stop Smoking Service, Burton-on-Trent, England

**Keywords:** Smoking cessation, Varenicline, Nicotine Replacement Therapy, Interactions, Gender differences

## Abstract

**Background:**

Smoking cessation services are available in England to provide assistance to those wishing to quit smoking. Data from one such service were analysed in order to investigate differences in quit rate between males and females prescribed with different treatments.

**Methods:**

A logistic regression model was fitted to the data using the binary response of self-reported quit (failed attempt = 0, successful attempt = 1), validated by Carbon Monoxide (CO) monitoring, 4 weeks after commencing programme. Main effects fitted were: client gender; age; region; the type of advisory sessions; and pharmacotherapy, Nicotine Replacement Therapy (NRT) or Varenicline. A second model was fitted including all main effects plus two-way interactions except region. These models were repeated using 12-week self-reported quit as the outcome.

**Results:**

At 4 weeks, all main effects were statistically significant, with males more likely (odds ratio and 95 % CI, females v males = 0.88 [0.79–0.97]), older smokers more likely (adjusted odds ratios [OR] and 95 % confidence interval [CI] respectively for groups 20–29, 30–49, 50–69 and 70+ vs 12–19 age group: 1.79 [1.39–2.31], 2.12 [1.68–2.68], 2.30 [1.80–2.92] and 2.47 [1.81–3.37] and for overall difference between groups, χ^2^(4) = 53.5, *p* < 0.001) and clients being treated with Varenicline more likely to have successfully quit than those on NRT (adjusted OR and 95 % CI for Varenicline vs NRT = 1.41 [1.21–1.64]). Statistically significant interactions were observed between (i) gender and type of counselling, and (ii) age and type of counselling. Similar results were seen in relation to main effects at 12 weeks except that type of counselling was non-significant. The only significant interaction at this stage was between gender and pharmacotherapy (adjusted OR and 95 % CI for females using Varenicline versus all other groups = 1.43 [1.06–1.94]).

**Conclusion:**

Gender and treatment options were identified as predictors of abstinence at both 4 and 12 weeks after quitting smoking. Furthermore, interactions were observed between gender and (i) type of counselling received (ii) pharmacotherapy. In particular, the quit rate in women at 12 weeks was significantly improved in conjunction with Varenicline use. These findings have implications for service delivery.

## Background

Smoking is a major cause of mortality and ill health worldwide [1]. Despite well-known associations with cancer of the lungs and other organs [2], and cardiovascular disease [3], the number of smokers in populations across the world remains high. According to the World Health Organization (WHO), 21 % of the global population aged 15 years and above smoke tobacco, and men smoke at five times the rate of women; 36 % vs. 7 % respectively [4]. Healthcare systems devote significant effort to promoting smoking cessation given the potential health benefits to the population, potential reduction of health service utilisation and saving of health expenditure due to morbidity attributable to smoking. This is important in the context of increasing pressures on healthcare budgets [5]. In the UK, smoking cessation support is provided to anyone wishing to quit through a number of specialised evidence-based services, generally known as “Stop Smoking Services” [6]. These services adhere to national guidelines provided by the National Centre for Smoking Cessation and Training [7], and National Institute for Health and Care Excellence [8], ensuring provision of effective and cost effective interventions. A client accessing these services is assisted by qualified smoking cessation advisers, who utilise behavioural change techniques in combination with tailored pharmacotherapy.

Smoking cessation services, or components of them, may not work equally across the target population. A number of studies have identified gender differences in relation to smoking quit rates. However, no universal consensus emerges in relation to the nature of this difference. Some studies report a statistically higher success rate for males [9–12], some suggest women are more likely to succeed in quitting [13], and in others the picture is inconclusive [14–16]. This lack of consistency may be related to differences between the study populations and smoking cessation interventions investigated. Different patterns are seen between male and female smokers in terms of behavioural patterns underlying smoking habit [9] and personal characteristics [17] as well as use and experience of smoking cessation services [18].

In the UK, three pharmaceutical interventions are currently offered as part of the smoking cessation service programme: Nicotine Replacement Therapy (NRT), Varenicline (Champix) and Bupropion (Zyban). All of these have been shown to enhance smoking quit rates relative to placebo/no treatment, with the best figures generally being seen in relation to Varenicline use [19–21] and no consistent difference emerging between Bupropion and NRT when considering results from different studies [22].

A few studies have looked into the relative benefits of pharmaceutical interventions in men and women. Amongst these, there is some evidence for the presence of interactions between gender and pharmaceutical agents that are available in the UK [10, 23–25] and also Topiramate in the United States [26].

The use and effect of smoking cessation services among different demographic subgroups of the population is not currently well understood. However, the characteristics of different subgroups is potentially important as a basis for adapting treatment programmes to meet the needs of different groups and optimising smoking quit rates across the population as a whole.

This study utilised a large dataset recorded by Quit-51 [27], one of a number of smoking cessation service providers in England. The aim was to assess (i) whether there is a difference in quit rate between males and females, (ii) whether there is a difference between males and females from different pharmaceutical interventions in the endeavour to quit smoking, and (iii) whether other factors influence outcomes for men and women and the benefit obtained from pharmaceutical interventions.

## Methods

Data were provided by Quit-51 based on clients attempting to quit smoking in four regions in England where the service operates (East Sussex, Sandwell, Walsall and Worcestershire). Quit-51 record a number of metrics on clients using the service, including individual-level information such as age and gender (male/female) of the client and details of the programme followed (treatment prescribed, the venue chosen for advisory sessions, number of sessions attended etc.). For analytical purposes, information was extracted on client gender, age, pharmacotherapy used (where information was available), the type of counselling taken up (one-to-one counselling, group sessions etc.) and various measures of quit success/failure.

The original dataset comprised a total of 7581 records on 6614 clients using the Quit-51 service across the four regions mentioned above between 15 March 2013 and 21 September 2015 (with reference to quit date). There was a small degree of replication, i.e. clients who used the system more than once. Records were removed where (i) the client was recorded as having been prescribed Bupropion for pharmacotherapy, *n* = 38; (ii) where more than one pharmaceutical treatment was recorded, *n* = 268; (iii) where the recorded age was less than 12 or greater than 93 or not recorded, *n* = 26. Restrictions (i) and (ii) were applied because the sample sizes were too low to make a meaningful comparison with the other treatments. Restriction (iii) was applied as ages recorded outside the range 12 – 93 years were considered likely to be erroneous. Applying the above criteria reduced the dataset from 7581 to 6959 records. In terms of replication, 5399 individuals appeared in the dataset once and 673 appeared more than once.

An initial model was fitted in which the response variable was a self-reported quit 4 weeks after the initial quit date supported by a blood Carbon Monoxide (CO) reading below 10 parts per million, in accordance with the Russell standard [28]. Quit status was recorded as “Y” (abstinent at stated time), “N” (smoking again at stated time), “L” (lost to follow-up) and in some cases the field was empty. Both “L” and missing cases were treated as unsuccessful quit attempts. Because of the binary nature of the outcome, analyses were carried out using logistic regression [29]. Although a degree of replication was present in terms of some clients appearing in the dataset more than once, data were treated as independent in analysis as this was a relatively rare phenomenon and considered unlikely to significantly bias statistical inference.

Random variation in the response was modelled with a Bernoulli distribution and dispersion fixed at unity. Explanatory variables fitted were: (i) gender, (ii) age, (iii) region, (iv) type of counselling, and (v) pharmacotherapy. A second model was fitted including, in addition to the above, all two-way interactions except those involving region. A backwards stepwise regression approach was applied removing interactions where statistical significance, as measured by the corresponding *p*-value, was above an *a priori* threshold of 5 % until only significant two-way interactions remained in the model.

Adjusted odds ratios [30] and corresponding 95 % confidence intervals (CI) for key main effects and interactions are presented. Statistical inference was based on Chi-squared tests when considering the overall effect of a variable. With respect to the interaction model, only statistically significant two-way interactions are presented.

Similarly, the main effect and interaction models were fitted to the data using the 12-week self-reported quit as response variable. All analyses were carried out in Genstat [31].

## Results

Frequencies and percentages are presented for each category of each variable for the dataset of 6959 records in Table 1. Similarly, the frequencies and percentages of male and female records across levels of all other variables in the models are given in Table 2. More than half the records (54.2 %) came from East Sussex and about a third (33.5 %) from Sandwell. In terms of pharmacotherapy, NRT was much more frequently used than Varenicline (87.4 % vs. 12.6 % records for NRT and Varenicline respectively) and there were somewhat more female users of the system (56.1 % vs. 43.9 % female and male records respectively). Heterogeneity in frequency of service use between males and females was present across different age groups with a high frequency of females users in the 20–29 year age group (63.8 % records in this age group from female clients) and a correspondingly low percentage amongst the over 70s (49.3 % records). The percentage of females in the Varenicline group was somewhat lower than in the sample as a whole (53.0 % of the subgroup prescribed Varenicline were female vs. an overall average of 56.1 %). Other observed differences are likely to have been influenced by low sample size and thus greater error around the mean.

**Table 1:** Frequencies (number of observations and %) across levels of key variables, *N* = 6959

Variable	Category	*N* (%)
Region^a^	East Sussex (1/1/14 to 21/9/15)	3773 (54.2 %)
	Sandwell (15/3/13 to 18/9/15)	2331 (33.5 %)
	Walsall (15/4/14 to 30/8/15)	140 (2.01 %)
	Worcestershire (14/4/13 to 30/8/15)	715 (10.3 %)
Sex	Male	3055 (43.9 %)
	Female	3904 (56.1 %)
Age	12–19	426 (6.12 %)
	20–29	1041 (15.0 %)
	30–49	2963 (42.6 %)
	50–69	2158 (31.0 %)
	70+	371 (5.33 %)
Counselling	One-to-one	5666 (81.5 %)
	Drop-in	350 (5.03 %)
	GP	326 (4.69 %)
	Pharmacy	207 (2.98 %)
	Nurse	165 (2.37 %)
	Telephone	185 (2.66 %)
	Other	57 (0.82 %)
Treatment	NRT	6085 (87.4 %)
	Varenicline	874 (12.6 %)

^a^Dates for which data were available for different regions are included in brackets

**Table 2:** Breakdown (frequency and %) of female records across levels of key variables

Variable	*N* (%)	% Females (mean = 56.1 %)
Region		
East Sussex	3773 (54.2 %)	56.8 %
Sandwell	2331 (33.5 %)	55.7 %
Walsall	140 (2.01 %)	42.1 %
Worcestershire	715 (10.3 %)	56.6 %
Age		
12–19	426 (6.12 %)	53.8 %
20–29	1041 (15.0 %)	63.8 %
30–49	2963 (42.6 %)	55.4 %
50–69	2158 (31.0 %)	55.0 %
70+	371 (5.3 %)	49.3 %
Counselling		
One-to-one	5666 (81.5 %)	56.9 %
Drop-in	350 (5.03 %)	43.7 %
GP	326 (4.69 %)	53.7 %
Pharmacy	207 (2.98 %)	54.1 %
Nurse	165 (2.37 %)	52.7 %
Telephone	185 (2.66 %)	58.4 %
Other	57 (0.82 %)	71.9 %
Treatment		
NRT	6085 (87.4 %)	56.5 %
Varenicline	874 (12.6 %)	53.0 %

### Four-week quit models

All variables fitted as part of the main effects model were statistically significant (Table 3). Males were found to have a better chance of quitting than females after 4 weeks. Elsewhere, the data show a progressive increase in quit rate with age, although this did not appear to happen in a linear fashion. In particular, we see a disproportionate jump in the quit rate going from the 12 to 19 year age group (34.7 %) to the 20–29 year group (53.1 %) and this is reflected in the adjusted odds ratio (1.79, 95 % CI = 1.39 to 2.31). Significant variation was present between different types of counselling with respect to successful 4-week quits. One-to-one counselling was associated with a high quit rate (significantly higher than both Pharmacy support and Nurse support categories). Finally, we see a higher rate of cessation among smokers using Varenicline as compared to NRT (63.6 % v 54.9 % respectively, adjusted odds ratio = 1.41, 95 % CI = 1.21 to 1.64).

**Table 3:** GLM model for 4-week validated quit; adjusted odds ratios with 95 % confidence intervals (CI) and significance results

Variable	Level	Quit rate – n/N (%)	Odds ratio (95 % CI)	Wald (d.f.)	*p*-value
Sex				6.7 (1)	0.01
	Male	1763/3055 (57.7 %)	1		
	Female	2133/3904 (54.6 %)	0.88 (0.79–0.97)		0.03
Region				192.9 (3)	<0.001
	East Sussex	2431/3773 (64.4 %)	1		
	Sandwell	1069/2331 (45.9 %)	0.49 (0.44–0.55)		<0.001
	Walsall	57/140 (40.7 %)	0.38 (0.27–0.54)		<0.001
	Worcestershire	339/715 (47.4 %)	0.48 (0.40–0.57)		<0.001
Age				53.5 (4)	<0.001
	12–19	148/426 (34.7 %)	1		
	20–29	553/1041 (53.1 %)	1.79 (1.39–2.31)		<0.001
	30–49	1690/2963 (57.0 %)	2.12 (1.68–2.68)		<0.001
	50–69	1275/2158 (59.1 %)	2.30 (1.80–2.92)		<0.001
	70+	230/371 (62.0 %)	2.47 (1.81–3.37)		<0.001
Counselling				71.7 (6)	<0.001
	One-to-one	3315/5666 (58.5 %)	1		
	Drop-in	148/350 (42.3 %)	1.14 (0.88–1.47)		0.4
	GP	155/326 (47.5 %)	0.89 (0.70–1.14)		0.2
	Pharmacy	82/207 (39.6 %)	0.55 (0.41–0.73)		<0.001
	Nurse	64/165 (38.8 %)	0.31 (0.22–0.42)		<0.001
	Telephone	108/185 (58.4 %)	1.01 (0.75–1.37)		0.6
	Other	24/57 (42.1 %)	0.55 (0.32–0.94)		0.02
Treatment				19.0 (1)	<0.001
	NRT	3340/6085 (54.9 %)	1		
	Varenicline	556/874 (63.6 %)	1.41		<0.001

In terms of interactions, the 4-week quit model retained the following two associations: (i) gender and counselling, and (ii) age and counselling (Table 4). With respect to sex and counselling, the odds ratios for the interactions between women and Pharmacy Support, Nurse Support, and Other Support were relatively large, although the number of clients in the Other support group was low making reliable estimation difficult. In terms of age and counselling, the odds ratios for all age groups above the baseline of 12–19 years in conjunction with GP Support were all greater than unity.

**Table 4:** Adjusted odds ratios and 95 % confidence intervals for combinations of significant interactions from model where response is 4-week validated quit (statistically significant ratios presented in bold)

	One-to-one	Drop-in	GP	Pharmacy	Nurse	Telephone	Other
Interaction 1: Gender• Counselling (χ^2^ = 15.1, d.f. = 6, *p* = 0.02)							
Sex							
Male	1	1	1	1	1	1	1
Female	1	1.23 (0.78–1.94)	0.82 (0.51–1.29)	**2.04 (1.11–3.75)**	1.84 (0.96–3.55)	1.02 (0.53–1.96)	**5.10 (1.27–20.55)**
Interaction 2: Age Group• Counselling (χ^2^ = 42.5, d.f. = 24, *p* = 0.011)							
Age							
12-19	1	1	1	1	1	1	1
20–29	1	1.24 (0.58–2.64)	78.4 (0.1–71986)	**0.1 (0.02–0.38)**	0.4 (0.02–8.31)	6.7 (0.64–70.74)	2.1 (0.23–19.23)
30–49	1	0.77 (0.43–1.39)	109.6 (0.12–98732)	**0.11 (0.03–0.44)**	0.29 (0.02–4.93)	2.73 (0.28–26.74)	0.55 (0.09–3.17)
50–69	1	0.90 (0.41–1.97)	76.2 (0.68–68671)	**0.1 (0.02–0.30)**	0.29 (0.02–5.02)	1.78 (0.18–17.63)	0.14 (0.02–1.33)
70+	1	48.4 (0.04–54871)	139.1 (0.14–136710)	**0.11 (0.02–0.67)**	0.34 (0.01–8.35)	0.18 (0.01–3.87)	0.83 (0.03–23.18)

### Twelve-week quit models

Results from the main effects model in relation to 12-week self-reported quit (Table 5) generally corroborated those above although the overall quit rate was lower (27.3 % compared to 56.0 % for 4-week quit). The difference between Varenicline and NRT was more pronounced after 12 weeks (adjusted odds ratio for Varenicline v NRT = 2.87, 95 % CI = 2.46 to 3.35 at 12 weeks as compared to 1.41 after 4 weeks). The magnitude of the difference between males and females was also slightly higher. Age differences followed the same general pattern with the difference between the older age groups in relation to the 12–19 year olds being greater when analysed at 12 weeks.

**Table 5:** GLM model for 12-week self-reported quit; adjusted odds ratios with 95 % confidence intervals (CI) and significance results

Variable	Level	Quit rate – n/N (%)	Odds ratio (95 % CI)	Wald (d.f.)	*p*-value
Sex				8.9 (1)	0.003
	Male	905/3055 (29.6 %)	1		
	Female	996/3904 (25.5 %)	0.84 (0.76–0.94)		0.003
Region				334.3 (3)	<0.001
	East Sussex	702/3773 (18.6 %)	1		
	Sandwell	913/2331 (39.2 %)	3.28 (2.89–3.73)		<0.001
	Walsall	37/140 (26.4 %)	1.50 (1.01–2.22)		0.04
	Worcestershire	249/715 (34.8 %)	2.29 (1.89–2.77)		<0.001
Age				82.7 (4)	<0.001
	12–19	89/426 (20.9 %)	1		
	20–29	211/2041 (20.3 %)	1.47 (1.08–2.01)		0.015
	30–49	829/2963 (28.0 %)	2.23 (1.68–2.95)		<0.001
	50–69	661/2158 (30.6 %)	2.72 (2.04–3.63)		<0.001
	70+	111/371 (29.9 %)	3.18 (2.22–4.56)		<0.001
Counselling				11.8 (6)	0.07
	One-to-one	1479/5666 (26.1 %)	1		
	Drop-in	130/350 (37.1 %)	1.43 (1.09–1.87)		0.01
	GP	125/326 (38.3 %)	1.05 (0.81–1.36)		0.7
	Pharmacy	68/207 (32.9 %)	1.17 (0.86–1.59)		0.3
	Nurse	35/165 (21.2 %)	0.80 (0.54–1.19)		0.3
	Telephone	53/185 (28.6 %)	1.13 (0.81–1.59)		0.5
	Other	11/57 (19.3 %)	0.57 (0.28–1.17)		0.1
Treatment				175.8 (1)	<0.001
	NRT	1513/6085 (24.9 %)	1		
	Varenicline	388/874 (44.4 %)	2.87 (2.46–3.35)		<0.001

**Fig. 1:**
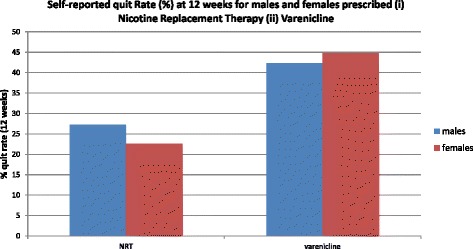
Self-reported quit Rate (%) at 12 weeks for males and females prescribed (i) Nicotine Replacement Therapy (ii) Varenicline

The only significant interaction was that between gender and pharmacotherapy (χ^2^ = 5.3, d.f. = 1, *p* = 0.02). This was reflected in an adjusted odds ratio of 1.43 (95 % CI = 1.06 to 1.94) with respect to females using Varenicline and the effect is clearly demonstrated by the raw data (Fig. 1).

## Discussion

The results of this analysis afford an insight into the effects of smoking cessation services and its components among men and women in a real-world setting. Much of the existing evidence for pharmaceutical interventions in smoking cessation comes from randomised controlled trials (RCT), demonstrating efficacy in ideal circumstances which may be significantly different to those in day to day practice. Real world studies complement RCTs and add to the evidence base for service delivery mechanisms.

Despite the presence of missing data in relation to some key fields, the available dataset was nonetheless large (*N* = 6959). The possibility of misrecording across different variables cannot be discounted. However, the size of the dataset is likely to provide a safeguard against bias arising from any resulting extra noise assuming such misrecording was rare and not systematic.

Differences emerge between men and women with respect to patterns in the use of smoking cessation services. Overall, there are more instances of women registered with the system than men (56.1 % v 43.9 % respectively). This may in part be a result of greater general engagement with health services by women as reported elsewhere [32].

In terms of age, women are especially well represented in the 20–29 year age group. It is possible to speculate that this may be related to a desire to quit smoking among women of child bearing age, who may be considering having a family. Amongst the over 70s, the number of males and females is almost the same. This may be a result of disproportionate prevalence of long-term complications of smoking, such as Chronic Obstructive Pulmonary Disease (COPD), between men and women in this age group given historically higher smoking rates in men.

All main effects were significant predictors of 4-week CO-validated quit success. The finding that males are more likely to quit has been observed elsewhere. In part, this may be explicable in terms of a perceived connection between smoking and weight loss potentially making more women reluctant to quit [33, 34]. The size of the difference reported here, whilst small in statistical terms, nonetheless translates to a high number of additional quitters when considered on a national scale assuming the generalisability of the result.

We see a clear increase in quit rate with age at both 4 and 12 weeks. Improved quit rates with increasing age have been observed elsewhere [35] although the current work highlights particularly poor success rates amongst the teenage cohort, possibly connected to lower health concerns within this age group. It is understood that quitting at an early age, e.g. before 35 [36], can almost completely negate the reduction in life expectancy attributable to cigarette smoking, thus it is important to achieve as high rates in this subgroup as possible. Despite the low rate of cessation observed here amongst teenagers, studies have nonetheless shown smokers in this age group to be responsive to cessation programmes [37]. More research is needed to better understand factors likely to enhance quit rates amongst early age smokers that can be incorporated into quit programmes.

The results here support the finding observed elsewhere that Varenicline is a more effective aid to smoking cessation than NRT [38]. In accordance with earlier findings, this effect was more pronounced after 12 weeks, which suggests longevity in the action of this treatment [39]. The reason for this may be related to cognitive deficits during nicotine deprivation [40]. The decision to make Varenicline more widely available for the purposes of smoking cessation is likely to depend on whether the treatment is perceived as cost effective and ‘good value for money’, given wider pressures on health service budgets. Different modelling studies indicate Varenicline is cost-effective relative to other treatments [41, 42]. Contraindications and side-effect profiles also need to be considered in making decisions on prescription at an individual level [43].

The outcomes considered here were on a relatively short timescale (4 and 12 weeks) and the importance of these results depends on whether these trends persist over a longer period (e.g. 12 months plus). Successful smoking cessation at 12 weeks has been shown to be a good indicator of long-term or permanent cessation [44, 45]. In order to achieve permanent abstinence from smoking many clients may require long term treatment with pharmacotherapy which implies additional cost [44].

Significant differences between the various types of counselling are somewhat difficult to interpret in light of the fact that the majority of clients take up one-to-one advice in this cohort. One-to-one advice is associated with a higher than average quit rate with respect to 4-week CO-validated quit (58.5 % v overall average of 56.0 %) but a lower than average rate in relation to 12-week self-reported quitting (26.1 % v overall average of 27.3 %). It is difficult to identify consistent patterns in both 4-week CO-validated and 12-week self-reported quit rates and this is an area which requires further investigation.

The introduction of smoking cessation programmes with tailored pharmacotherapy remains a relatively recent innovation and it is the case that many smokers will try to quit unassisted, not least in parts of the world where such programmes are in their infancy. It would therefore be of interest to compare cohorts of quitters both inside and outside of the service. By the nature of the current data, it was not possible to investigate this question (by definition, clients in the dataset were registered with a cessation service). There is ample evidence of the efficacy of individual aspects of the service, e.g. pharmacotherapy against placebo [19] and counselling sessions [37], but it would be valuable to see how these effects operate together in a real-world setting.

The significant interaction between client gender and pharmacotherapy after 12 weeks indicates that women in particular benefit from Varenicline use, notwithstanding lower overall quit rates in relation to men. This accords with results from meta-analysis of clinical trials [46] and suggests that efficacy in these trials is mirrored in real life.

The prescription of Varenicline for female quitters is complicated by concerns regarding its suitability in pregnancy [47–49]. Whilst NRT was much more frequently prescribed by smoking cessation services than Varenicline in this sample (87.4 % v 12.6 % respectively), men are more likely to receive Varenicline than women. Again, this may in part be due to concerns over side-effects from using Varenicline during pregnancy.

## Conclusions

These results from analysis of data on patients using a smoking cessation service provide an insight into the effects of interventions in a real world setting. Under the current prescription, we see that males using the service are more likely to succeed in quitting. Individual performance is also influenced by age and type of counselling provided. Varenicline support was found to enhance the chance of a successful quit in relation to NRT and this benefit was found to be particularly pronounced for females when quit success was measured at 4 weeks. Given that women tend to receive Varenicline as an aid to smoking cessation less than men, this is an imbalance which could be redressed in future.

## Abbreviations

CI: Confidence interval
CO: Carbon monoxide
GLM: Generalised linear model
NRT: Nicotine replacement therapy
OR: Odds ratio
