# Erratum to: Gender difference and effect of pharmacotherapy: findings from a smoking cessation service

**DOI:** 10.1186/s12889-017-4205-z

**Published:** 2017-04-04

**Authors:** N. J. Walker, H. C. van Woerden, V. Kiparoglou, Y. Yang, H. Robinson, E. Croghan

**Affiliations:** 1 Oxford Biomedical Research Centre, Churchill Hospital, Oxford, England; 2 Institute of Primary Care & Public Health, Cardiff University, Cardiff, Wales; 3 Centre for Health Sciences, University of the Highlands and Islands, Inverness, Scotland; 4 Nuffield Department of Primary Care Health Science, University of Oxford, Oxford, England; 5 Quit 51 Stop Smoking Service, Burton-on-, Trent, England

## Erratum

Following publication of this article [1], it has come to our attention that a line has been omitted from the acknowledgements. Could you please add the line, “This research was supported by the NIHR Oxford Biomedical Research Centre”.
